# A Case of Placenta Prævia

**Published:** 1869-12

**Authors:** J. P. Hamilton

**Affiliations:** Darlington, Wisconsin


ARTICLE IX.—*A Case of Placenta Prævia.* By J. P. HAMILTON, M.D., Darlington, Wisconsin.

THE following case may be of interest to your readers. It was one of great interest to me, it being the first of the kind that has occurred in my practice, extending over twenty-eight years.

On the sixth day of August last I was called to see a Mrs. G., of this village, a very intelligent lady, pregnant with her seventh child, and a week or ten days over eight months advanced. She informed me that about four o'clock that morning she was attacked with uterine hæmorrhage, and that it had continued, though slightly, up to the time of my visit, which was about seven, A.M. The hæmorrhage came on, according to her statement, without any known cause or pain, and with a gush. With this statement, I did not at once suspect the kind of case I had to treat. I had attended Mrs. G. in her two previous confinements, in both of which there were no untoward symptoms. I recommended her to keep quiet, and left her four powders, containing acet., plumb. and pulv. opii, to be taken every hour till the hæmorrhage was controlled. The case went on to the tenth. About the same time in the morning I was called. I found the flooding had returned. The nurse informed me that she had given her but two of the powders I had left at my previous visit; that the flooding had ceased after giving the two, and Mrs. G. had been quite comfortable till four o'clock this morning. I recommended her to take the balance of the powders, and quietness. I heard nothing further from the case till the fifteenth. At seven, A.M. I was called in haste; found my patient flooding alarmingly; her face blanched, pulse weak and fluttering. Her husband (who is a very intelligent gentleman) stated she had lost considerable blood, and appeared very much alarmed. I immediately made a vaginal examination; found the os fully dilated, and, on further examination, I found I had a case of placenta prævia, the centre of the placenta placed over the os, and the patient's life ebbing out every moment. Whatever I was to do to save my patient must be done immediately. Having a phial of chloroform in my pocket, I poured a small quantity on a handkerchief, handed it to her husband, requested him to apply it to her mouth and nose, and without changing her position, I introduced my hand into the mouth of the uterus, perforated the centre of the placenta and membranes, carried my hand up to the presenting part of the child (which was the head), pushed up my hand till I found a foot, which I brought down, and delivered the child as far as the umbilicus. The hæmorrhage having ceased at this stage of the delivery, I waited till the uterus had pretty firmly contracted. I delivered the balance of the child without very much loss of blood. The child was a female, of small size, and still-born; but a very little effort sufficed to resuscitate it. The placenta came away in a short time. By administering stimulants I had the satisfaction to see my patient rally, and had as good recovery (excepting debility from the loss of blood) as after any of her previous confinements.

Now, Mr. Editor, I don't claim that there is any thing unusual in the management of the above case, but one thing, and that is the perforation of the placenta, and extracting the child through the rent of the same. All the standard works that I have examined are opposed to that, as far as I have investigated the subject. They direct the student to detach the edge of the placenta and rupture the membranes and version. I am satisfied, had I followed this instruction, I should have lost valuable time and my patient; for time was a very important consideration, as far as my case was concerned. Prof. Dewees is considered very good authority in such matters. He gives eight reasons why it should not be done. As far as one case goes, it proves the utter fallacy of his reasoning. My hand passed through the placenta, using but very little force, and the child passed through the rent made by the hand without any difficulty.

I am satisfied that I succeeded in delivering my patient in a much shorter time than by any other course I could have adopted, and that, in my case, as I have stated, was a *desideratum*.

Should I ever have a similar case, I should treat it as I have this, believing it to be safer to mother and child, by being sooner performed.

